# Effectiveness of the addition of therapeutic alliance with minimal intervention in the treatment of patients with chronic, nonspecific low back pain and low risk of involvement of psychosocial factors: a study protocol for a randomized controlled trial (TalkBack trial)

**DOI:** 10.1186/s13063-017-1784-z

**Published:** 2017-01-31

**Authors:** Felipe Ribeiro Cabral Fagundes, Caique de Melo do Espírito Santo, Francine Mendonça de Luna Teixeira, Thaís Vanelli Tonini, Cristina Maria Nunes Cabral

**Affiliations:** 1 Master’s and Doctoral Programs in Physical Therapy of the Universidade Cidade de Sao Paulo, Rua Cesário Galeno 475, Tatuapé, São Paulo, SP CEP: 03071-000 Brazil; 2 Physical Therapy Department of the Universidade de Taubaté, Av. Marechal Arthur Costa e Silva 1055, Centro, Taubaté, SP Brazil

**Keywords:** Low back pain, Empathy, Subgroups, Minimal intervention

## Abstract

**Background:**

The stratified model of care has been an effective approach for the treatment of low back pain. However, the treatment of patients with low risk of psychosocial-factor involvement is unclear. The addition of the therapeutic alliance to a minimal intervention may be an option for the treatment of low back pain. This paper reports on the rationale, design and protocol for a randomized controlled trial with blind assessor to assess the effectiveness of the addition of therapeutic alliance with minimal intervention on pain and disability in patients with chronic, nonspecific low back pain.

**Methods:**

Two hundred and twenty-two patients with chronic, nonspecific low back pain and low risk of involvement of psychosocial factors will be assessed and randomly allocated into three groups (*n* = 74 patients per group). The Positive Therapeutic Alliance group will receive counseling and guidance with an emphasis on therapeutic alliance and empathy. The Usual Treatment group will receive the same information and counseling with limited interaction with the therapist. The Control group will not receive any intervention. The treatment will be composed by two intervention sessions with a 1-week interval. A blinded assessor will collect the following outcomes at baseline, 1 month, 6 months and 12 months after randomization: pain intensity (Pain Numerical Rating Scale), specific disability (Patient-specific Functional Scale), general disability (Oswestry Disability Index), global perceived effect (Global Perceived Effect Scale), empathy (Consultation and Relational Empathy Measure), credibility and expectations related to treatment. The analysis will be performed using linear mixed models.

**Discussion:**

This will be the first study to understand the effect of combining enhanced therapeutic alliance to a treatment based on counseling, information and advice (minimal intervention). The addition of the therapeutic alliance to minimal intervention may improve the treatment of chronic, nonspecific low back pain.

**Trial registration:**

ClinicalTrials.gov, NCT 02497625. Registered on 10 July 2015.

**Electronic supplementary material:**

The online version of this article (doi:10.1186/s13063-017-1784-z) contains supplementary material, which is available to authorized users.

## Background

Low back pain remains a global public health problem [1]. It is considered to be the sixth leading cause of disability-adjusted life years among more than 200 health conditions [1, 2]. Despite the increase in quantity and quality of research in recent decades, the available treatments for low back pain tend to produce minor or moderate effects. These effects are positive in the short term and only some of them maintain long-term improvements [3]. The high prevalence of low back pain makes impossible the use of treatment strategies that demand high resources for all patients [4]. Studies have shown that a small group of patients who develop chronic pain are responsible for most of the treatment-related costs [5, 6]. Thus, it is important to identify patients who would benefit from a specific treatment from those who would show little benefit or even worsening of symptoms [4].

Low back pain is an ideal condition for the stratification of treatment subgroups since it is a condition that affects a heterogeneous population, presenting prognostic variation and relying on different diagnostic options and available treatments [4, 7, 8]. Stratification based on the prognosis of the clinical condition is a form of classifying low-back-pain patients in subgroups and it is based on the risk of developing persistent pain and disability related to psychosocial factors [4]. Psychosocial factors (e.g., depression, stress, aspects related to work and fear of movement [9]) are predictive for the occurrence of new episodes and the long-term persistence of pain and disability [8, 10–12]. Treatment in subgroups may also facilitate the clinical decision-making process and guide appropriate treatment for these patients [4]. Moreover, it may assist therapeutic decision-making, maximize treatment benefits, reduce risks and increase health system efficiency [8, 13].

Recent studies have shown the cost-effectiveness of the treatment based on the chronic low back pain prognosis in patients with high and medium risks of developing chronic problems related to psychosocial factors [8, 14]. Positive results were associated with decreased pain and disability, in addition to reduction in cost and work absenteeism [7, 14]. However, the results of a recent randomized controlled trial showed no significant differences for patients with low-risk of involvement of psychosocial factors when compared the usual treatment and targeted treatment based on subgroups [15]. These negative results may be explained by the fact that patients already presented low levels of pain and disability and by the use of questionnaires with low responsiveness for low-risk patients [16]. Disability questionnaires frequently used to evaluate patients with low back pain are not sensible to detect a clinical change in patients with low level of disability. For this kind of patients, it is recommended to use specific questionnaires of disability [16]. In the treatment of these low-risk patients it is recommended that information and guidance be provided on correct diagnosis, prognosis, symptoms, physical activity levels, return to work and disease severity, preferably at the first contact with the health professional [9, 17, 18]. Considering the good prognosis of low-risk patients, some studies suggest a minimal intervention approach (counseling sessions and positive information) [7, 17]. This approach might be a quick and low-cost treatment option to the health system. Furthermore, identifying appropriate treatment for each patient may prevent the unnecessary use of expensive or extended resources [4]. There is also the possibility of reducing the use of diagnostic procedures (imaging tests, for example), decreasing the number of consultations during the recovery process and consequently less use of financial resources. Thus, some studies emphasize the need to develop more effective treatment strategies for this patient subgroup [7, 8, 14]. One of the possibilities of the nonsignificant results deriving from minimal intervention with these patients is that patients with low risk of developing chronic problems have low to medium levels of pain and disability. Therefore, it is necessary to evaluate patients using measurement instruments that are more responsive to this clinical condition [16].

The extent of treatment effectiveness may be influenced by factors other than the intervention chosen. Contextual factors comprise the therapeutic effect of any intervention. These factors consist of a complex interaction between technical and communicative knowledge in addition to the therapist’s ability to meet the expectations of the patient during treatment [19]. Studies have shown that a good relationship between the therapist and patient is related to decreased pain level and disability as well as improved satisfaction with treatment [20, 21]. The therapeutic alliance may be defined as harmony or social connection between therapist and patient [22]. However, both the magnitude and extent of this association in musculoskeletal patients are unknown [21]. This gap in the medical literature reinforces the need for studies on this subject, especially in the treatment of patients with musculoskeletal complaints. Emerging evidence indicates that the degree and quality of the interaction between clinician and patient is important because it influences the magnitude of the active treatment, as well as the degree of placebo effect [21, 23, 24]. Although therapeutic alliance has been shown to produce therapeutic benefits in some areas, such as medicine and psychotherapy, there is little empirical support yet for this subject in physiotherapy [20, 21]. A recent systematic review showed a consistent pattern of positive therapeutic alliance correlated with improved pain, disability and treatment satisfaction in physical rehabilitation [21].

A recent study investigated the effects of increased therapeutic alliance and empathy in the treatment of patients with low back pain receiving active interferential current or a placebo. Patients were randomly allocated into four groups: (1) limited interaction plus active current, (2) limited interaction plus placebo, (3) increased interaction plus active current and (4) increased interaction plus placebo. The results showed clinical improvement associated with increased interaction between therapist and patient, which remained even with the application of placebo [25]. This study evaluated the effects only in the short term and did not classify patients into specific treatment subgroups. Therefore, the rationale of this study is based on emerging evidence of the importance of the therapeutic alliance in the clinical context [19, 21, 23–29].

Appropriate treatment for low-risk patients can provide important clinical benefits. It could be a fast and low-cost treatment option for the health system since it would help in identifying patients who do not require unnecessary or extensive assessment and treatment [4]. To date, there are no studies that have investigated the therapeutic alliance combined with minimal intervention in the treatment of patients with chronic, nonspecific low back pain and low risk of psychosocial-factor involvement. Therefore, the objective of this study is to evaluate the effectiveness of the addition of the therapeutic alliance with minimal intervention in the treatment of patients with chronic, nonspecific low back pain with a low risk of having psychosocial-factor involvement in their pain, specific and general disability, global perceived effect, empathy, credibility and expectation. The hypothesis of this study is that there will be a clinical benefit in pain, and especially in specific disability, 1 month after randomization into the group receiving treatment with the addition of the therapeutic alliance.

## Methods

### Study design

This is a three-arm randomized controlled trial with blinded assessor. The patients will be divided into three groups: a Positive Therapeutic Alliance (PTA) group, a Usual Treatment (UT) group and a Control group (CG). Table 1 details the study timeline and follows the style of the Standard Protocol Items: Recommendations for Interventional Trial (SPIRIT) guideline. The SPIRIT Checklist and figure for the study protocol are provided as Additional file 1 and Additional file 2. The World Health Organization Trial Registration Data Set is provided as Additional file 3.

**Table 1:** Timeline for the schedule of enrollment, interventions and assessments

Results	Recruitment	Before randomization	Post baseline (after first session)	Intervention period	Reassessment 1 month after randomization	Reassessment 6 months after randomization	Reassessment 12 months after randomization
Eligibility criteria	X						
Demographic data	X						
Informed consent	X						
Primary outcomes							
Pain intensity		X			X		
Specific disability		X			X		
Secondary outcomes							
Pain intensity						X	X
Specific disability						X	X
General disability		X			X	X	X
Global perceived effect		X			X	X	X
Additional outcomes							
Empathy			X				
Credibility			X				
Expectation			X				
Interventions							
Increased therapeutic alliance				X			
Usual treatment				X			
Control group				X			

### Sample size calculation

The sample size calculation was carried out to detect a difference of 1 point on the Pain Numerical Rating Scale (estimated standard deviation = 1.84) and 1 point on the Patient-specific Functional Scale (estimated standard deviation = 1.8). The calculations were performed considering an *α* = 0.05, a statistical power of 80% and a follow-up loss of 15%. Thus, this study will comprise a sample of 222 patients who will be divided into three treatment groups (*n* = 74 patients per group).

### Eligibility criteria

This study will be carried out with patients on the treatment waiting lists of two physical therapy teaching clinics in the city of Taubaté, São Paulo (approved by the Ethics Committee of Universidade Bandeirante Anhanguera n. 44720315.5.0000.5372). The patients will be invited personally by phone. This study will include patients who have had chronic, nonspecific low back pain for at least 3 months, aged between 18 and 80 years, and who are able to read and write in Portuguese and classified as low risk of having psychosocial factors involved [8] according to the Start Back Screening Tool (SBST-Brazil) questionnaire [30, 31]. Patients with a history of spinal surgery, serious spinal diseases, nerve root compromise, diseases associated with cognitive impairment confirmed by medical diagnosis, or pregnancy will be excluded.

### Procedures

Patients will be evaluated by a previously trained blinded assessor who will provide information on the procedures and criteria for study participation. The assessor will contact patients by phone to confirm their eligibility criteria and to fill out the SBST-Brazil questionnaire. In the case of acceptance of participation and compliance with the eligibility criteria, the patient will be forwarded to initial assessment.

The blinded assessor will collect data at the baseline utilizing a neutral communication style so as not to influence the therapeutic alliance involved in patient care. A coding system in follow-up assessments will be carried out to ensure blinding. After this initial procedure, eligible patients will be referred to the therapist responsible for the interventions. An independent researcher not involved in the recruitment and evaluation of patients will perform randomization using Microsoft Excel software random number generation. The secret allocation will be made using sequential, numbered, sealed opaque envelopes. Due to the nature of the study, it will not be possible to blind the therapist or patients. All researchers involved in the stages of this study will receive adequate training to carry out the activities. Patient recruitment will begin in September 2015 and the date of completion will be December 2016.

### Assessments

The elegibility criteria, collection of demographic and anthropometric data from the patients as well the outcomes will be conducted by a blinded assessor. The randomization to the treatments groups will be done before the initial evaluation. The assessor will be aware of the allocation only after data analysis.

All the questionnaires and scales used to assess primary and secondary outcomes have been translated and adapted into Brazilian-Portuguese and have shown adequate measurement properties. The primary outcomes of this study will be pain and specific disability after 1 month of randomization. The study’s secondary outcomes will be pain and specific disability 6 and 12 months after randomization; and general disability and perceived global effect 1, 6 and 12 months after randomization. Empathy, credibility and expectations related to treatment will be used as additional outcomes.

#### Demographic characteristics

The initial assessment information will include age, gender, height, weight, educational level and job information related to low back pain (pain location, duration, use of medication and previous treatments).

#### STarT Back Screening Tool

The SBST-Brazil questionnaire is a prognostic evaluation tool which aims to classify patients into low, medium or high risk of developing long-term pain or disability related to psychosocial factors [30, 31]. The questionnaire contains questions related to pain, disability, comorbidities, bothersomeness, pain catastrophizing, fear, anxiety and depression. For scoring and classification, the patient must choose between “agree” (1 point) or “disagree” (0 points) on the first eight questions. Question 9 contains five possible answers: not at all (0 points), slightly (0 points), moderately (0 point), very much (1 point) and extremely (1 point). The total score of the SBST-Brazil represents the sum of all questions and ranges from 0 to 9. Scores ≤3 points classify the patient as low risk. Scores ≥4 points indicate the need to examine the psychological subscale comprised of questions 5 to 9. Patients with scores ≤3 points on the psychological subscale are classified as medium risk and patients with scores ≥4 are classified as high risk. The SBST-Brazil was only used for screening and not as an outcome measure instrument.

#### Pain Numerical Rating Scale

The Pain Numerical Rating Scale is a scale of 11 points (0–10) that aims to measure the intensity of pain reported by the patient, where 0 = “no pain” and 10 = “pain as bad as could be” [32]. In this study patients will be asked about their pain intensity in the last 7 days.

#### Patient-Specific Functional Scale

The Patient-specific Functional Scale provides reliable measurements of limitations in specific tasks [32]. The patient is asked to choose three activities that they cannot perform or can perform with difficulty because of pain. Each activity is scored on a Likert scale that ranges from 0 (unable to perform the activity) to 10 (able to perform the activity at preinjury level). The final result is the average of the three scores from 0 to 10 points. High values indicate a lower degree of limitation in performing specific tasks [33, 34]. This questionnaire presents good responsiveness with patients with low functional limitation [16].

#### Oswestry Disability Index

The Oswestry Disability Index aims to assess disability associated with low back pain [35, 36]. This questionnaire consists of 10 items describing everyday situations that patients may find difficult to perform due to low back pain. Each of the 10 items is scored from 0 to 5, adding up to a maximum score of 50 points. The result is multiplied by 2 to obtain a percentage. From this obtained percentage it is possible to classify the patients as having: minimal disability (0% to 20%), moderate disability (20% to 40%), severe disability (40% to 60%), crippled (60% to 80%) and bedridden (80% to 100%).

#### Global Perceived Effect Scale

The Global Perceived Effect Scale seeks to evaluate clinical changes perceived by the patient by comparing the onset of symptoms to the current situation [32]. It is an 11-point grading scale (−5 to 5) wherein −5 = vastly worse; 0 = no change; and 5 = completely recovered. High scores indicate greater perceived recovery of the condition [32]. This scale is more responsive with patients who have low functional limitation [16].

#### Consultation and Relational Empathy – CARE

The Consultation and Relational Empathy (CARE) Measure is a questionnaire to assess patients’ perception of empathy and communication during consultation [37–39]. It consists of 10 questions answered according to a Likert scale of 1 to 5 (where 1 = poor and 5 = excellent). The final score is obtained by adding the questions’ scores, with a maximum score of 50 and a minimum score of 10 points. High values indicate greater empathy. Each question contains a “not applicable” answer; in this case, the item must be excluded and the result will be obtained from the average score of the questions answered [37]. The questionnaire will be applied by a blinded assessor after the first treatment session.

#### Expectation and credibility

Improvement expectation will be assessed by a numerical scale of 11 points, with 0 = “no expectation of improvement” and 10 = “the highest expectation of improvement” [40]. Credibility regarding the treatment will be evaluated by four questions. These questions include confidence-related issues that treatment can help to ease and deal with pain, confidence in recommending treatment to other patient and how much treatment makes sense to the patient. These questions will be scored according to a Likert scale ranging from 0 (no confident) to 6 (totally confident) [40]. Expectation and credibility will be evaluated after a week of randomization.

### Treatment protocol

Patients will be randomized into three groups: a Positive Therapeutic Alliance (PTA) group, a Usual Treatment (UT) group and a Control group (CG). Treatment sessions will be conducted face-to-face and individually with each patient. The treatment sessions will be delivered by two physical therapists with postgraduate qualifications and a completed Master’s degree in the research field.

The treatment protocol offered to the PTA and UT groups will be based on research that demonstrates the effectiveness of empathy and communication training with patients [26–28, 41]. Patients in the PTA and UT groups will receive two 60-min treatment sessions with a 1-week interval. The sessions will be held in an ordinary physical therapy office following the standards of comfort, organization and hygiene. The treatment protocol is initially structured in education and differentiation regarding the nonverbal behavior of the therapist and key issues related to the treatment. In order to ensure treatment fidelity, and to standardize the information that will be provided to patients in both groups, the therapists will follow a description of the relevant issues to be addressed during treatment (Tables 2 and 3). The treatment protocol recommendations were developed based on previous studies and designed to ensure the enhancement of the therapeutic alliance in one group and reduce it in the other [26–28, 41]. Considering the type of intervention, the therapists will not need any physical or informational materials to deliver the intervention. It is important to emphasize that the PTA and UT groups will receive the same informational content related to low back pain. The difference will be the form used to transmit this information.

**Table 2:** Topics to be addressed during therapy in Positive Therapeutic Alliance and Usual Treatment Groups [9, 27, 41–43, 47–54]

Addressed topic	Key issues to be discussed	Example sentences
Regarding low back pain	• Low back pain causes • Low back pain diagnosis • Low back pain classification • Prognosis • Low back pain natural history	“Permanent or severe back damages are rare” “It is really very difficult to damage your back” “Back pain is rarely caused by a serious illness” “Back pain prognosis is favorable”
Imaging tests	• Imaging findings in asymptomatic people • Normal degenerative signs	“About 60% of the asymptomatic population has some sort of degenerative sign” “There is nothing seriously wrong in your back”
Physical activity and Rest	• The importance of physical activity • Low back pain behavior	“The sooner you resume your activities the sooner you will get better” “Rest is not good for the back” “You may have some limitations, but the most important thing is to keep moving”
Pain control	• Medication • Available treatments • Self-care	“There are several steps you can take to help your back pain” “Physicians and physical therapists can help your back pain, but only you are able to take care of yourself”
Family and work, anxiety and stress-related factors	• The importance of family support • The importance of controlling anxiety and stress • The influence of these factors on back pain	“Gradually return to your social activities and work! The sooner you return, the less future problems you will have” “You really can help yourself!” “People who cope better with the pain return to their activities faster and better” “You will have good and bad days, this is normal!” “Although the natural history is positive, there are some factors that contribute to pain that becomes chronic, we will work on them”
Concepts of chronic pain	• Current concept of pain • Pain threshold • Central hypersensitivity	“Pain does not mean back injury” “Pain is a response to sensory stimuli, and it is not necessarily related to any tissue damage in the spine” “Pain can just be a misunderstanding of the body”
Encouragement ^51,55^	• Good prognosis: subgroup of low risk of involvement of psychosocial factors • Return to activities	“This information is taken from the best and most current research in the world” “Do not let your back pain take over your life, you are able to get through it”

**Table 3:** Guidelines for standard treatment sessions of the Positive Therapeutic Alliance Group [55–59]

Addressed aspect	Description	Key issues to approach
Appointment outset	A warming and caring reception	“Hello! I was waiting for you!” “Apart from the pain, are you all right today?”
Demographic questions	Show interest in getting to know the patient	“I think I know your family name…” “Tell me more about yourself”
Medical diagnosis	Ask about patient’s condition in an interested manner	“This part of the assessment interests me very much, tell me about your condition, what did the physician say?”
Primary complaint	Demonstrate interest in the current complaint	“In your daily routine, what bothers you the most? What is your primary complaint? Tell me so I can help you.”
Clinical history	Address issues about the patient’s condition (beginning, development, current condition) Inquire about medical history, involving other issues, not only low back pain	“First, I have a few questions that will help me better understand your pain”
Pain location	Investigate and demonstrate an effort to understand the patient’s pain complaint	“Use your hand to show me where your pain is” “From this point, does this pain go up/down or sideways?
Pain behavior	Investigate and demonstrate an effort to understand the patient’s pain complaint	“I am also interested in your personal symptoms” “Does this pain change during the day?” “Describe the pain to me” “Which activity of your daily life makes the pain worse?”
Primary limited daily activity	Question the patient demonstrating an interest in a way to encourage a return to activities	“How did this pain affect your lifestyle?”
Session closure	Reinforce positive messages	“You did very well today! And certainly you will be fine” “I understand that these chronic situations sometimes get out of our control, but I am sure you will do well” “No matter what happens. Try to be patient with yourself and look for positive things that are happening to you ”
Eye contact	Maintain eye contact while listening to the patient	-
Facial expressions in response to patients’ questions	Demonstrate emotions through facial expression Facilitate nonverbal communication through facial clues	-
Posture	Adopt a posture of attention, mutual respect and receptivity	-
Tone of voice	Use different tones of voice to emphasize the importance or the focus on a particular subject during treatment	-
Affection	Demonstrate the importance of patient participation in the therapeutic process Ask about emotional aspects	-
Understand the biopsychosocial context	Address issues related to work, family and social life	-
Shared decision making	Share decision-making regarding treatment Involve the patient in the healing context	“Shall we set some goals together in order to improve your condition?”
Demonstration of empathy	Reflect the emotions that patients present Create an environment of comfort and responsiveness	“Your back pain must be very hard for you” “I can put myself in your shoes and feel how that pain hinders you”
Concerning the treatment provided	Focus on information about the success of treatment, based on the best available scientific evidence	“The treatment being offered to you is scientifically proven to be the most suitable for your case”
Personal experience	Demonstrate successful experiences with similar cases	“The clinical experience I have had with this new treatment is promising! Often patients already feel much better soon after the treatment”

The patients in the PTA group will undergo an intervention based on treatment guidelines involving guidance and information related to a return to daily activities, advice on dealing with the pain and clear explanation of their signs and symptoms [9, 18, 42, 43]. The sessions will be structured with the intention of increasing therapeutic alliance and empathy, based on topics relating to the condition and behavior taken from *The Back Book* [43]. UT-group patients will also receive the same informational content on low back pain based on *The Back Book* [43]. Therapy sessions will be performed with limited interaction between patient and therapist and the information will be transmitted in a clear and straightforward manner [23]. Patients in the CG will not receive any treatment and will be instructed not to seek treatment for the first month after randomization. The treatment offered to the PTA or UT groups will be available for patients who are interested after a period of 1 year. Patients will not be limited in looking for other types of treatment after the first month and will be evaluated at baseline and during every reassessment. Since patients will be recruited from a waiting list and will receive the care offered in this study after 1 year, we believe that this conduct does not violate any ethical principles with these patients.

Patients who require additional interventions other than those offered by this study will be referred for treatment at teaching clinics of the participating universities. Patients’ use of medication will not be limited and will be controlled at each reassessment. Patients will be advised not to seek other treatment options during the intervention period. Patients will not receive any financial compensation for participation.

### Statistical analysis

A researcher not involved in data collection and without any conflict of interest will be responsible for monitoring data. The randomization process and blinding of patients and assessors will be audited monthly by an experienced researcher. All data will be entered twice before statistical procedures. A researcher who will receive the data in an encrypted format to ensure confidentiality will conduct the statistical analysis.

Linear mixed models will be used for primary analysis to verify the differences between the average effects of interventions in reassessments carried out 1 month, 6 months and 12 months after randomization. The differences between groups will be calculated by interaction terms of groups versus time. The time will be coded as a categorical variable (baseline, 1 month, 6 months and 12 months after randomization). No interim analysis will be performed. The process of analysis of treatment efficacy will follow the intention-to-treat analysis [44]. If a patient abandons treatment or refuses to answer any reassessment, the lost data will not be replaced. All statistical procedures will be performed with a significance level of 5% and through the SPSS 19 software for Windows.

## Discussion

The results of this study will help us to understand the effect of combining increased therapeutic alliance with a type of treatment based on advice, information and advice for patients with chronic low back pain and low risk of psychosocial-factor involvement. We believe that the UT group will present significantly better treatment results when compared to the CG. Regarding the PTA group, the study hypothesis is that: 1 month after randomization, there will be a significant clinical decrease in pain and especially in specific disability, when compared to the other two study groups. This assumption is based on recent studies, which have shown that the therapeutic alliance does in fact have a positive influence on clinical outcomes of patients with irritable bowel syndrome [23], rehabilitation needs [21] and chronic musculoskeletal disorders [29]. Treatment adherence appears to be directly influenced by patients’ trust and empathy with the health care professional. Thus, decreased specific disability may occur because the patient follows the guidelines provided by the health care professional with more confidence. Our research group expects to publish the results of this study in an internationally recognized journal and to release the spreadsheet with the data encoded during the first semester of 2018.

It is known that a minority of patients who develop chronic pain are responsible for most of the treatment-related costs [5, 6]. It is, therefore, important to identify whether patients at low risk of psychosocial-factor involvement respond positively to a minimal, effective and inexpensive treatment intervention. To date, this study is the largest randomized controlled trial involving the combination of therapeutic alliance with minimal intervention in patients with chronic, nonspecific low back pain. The results of this study may help the clinical practice of professionals dealing with patients with chronic, nonspecific low back. The health system can benefit from this type of intervention, avoiding excessive or unnecessary diagnostic investigations and treatments for these patients. This may lead to cost reduction regarding medication, diagnostic tests, consultations, treatments or absence from work. Furthermore, the health care system could provide access and opportunity for treatment for patients who require more attention to symptom evaluation and treatment (medium and high risk psychosocial-factor involvement). In conclusion, there might be the possibility to reduce waiting time for treatment, facilitating the flow and quality of primary care for low-risk low-back-pain patients.

## Trial status

Recruitment started in September 2015 and is expected to continue until December 2016.

## Additional files

Additional file 1: SPIRIT 2013 Checklist: Recommended items to address in a clinical trial protocol and related documents*. (DOC 121 kb)
Additional file 2: SPIRIT Figure with the timeline for the schedule of enrollment, interventions, and assessments. (DOC 56 kb)
Additional file 3: World Health Organization Trial Registration Data Set. (DOC 43 kb)

## Abbreviations

CARE: Consultation and Relational Empathy Measure
CG: Control group
PTA: Positive Therapeutic Alliance
SBST-Brazil: STarT Back Screening Tool – Brazilian-Portuguese version
UT: Usual Treatment
