# Supplementary material for: Effectiveness of the addition of therapeutic alliance with minimal intervention in the treatment of patients with chronic, nonspecific low back pain and low risk of involvement of psychosocial factors: a study protocol for a randomized controlled trial (TalkBack trial)
Source: Trials. 2017 Jan 31;18:49. doi: 10.1186/s13063-017-1784-z (PMC5282714; doi:10.1186/s13063-017-1784-z)
Supplement: Additional file 2: — SPIRIT Figure with the timeline for the schedule of enrollment, interventions, and assessments. (DOC 56 kb) [file 13063_2017_1784_MOESM2_ESM.doc]

Additional file 2 – SPIRIT Figure with the timeline for the schedule of enrollment, interventions, and assessments.

|  | **STUDY PERIOD** | | | | | |
| --- | --- | --- | --- | --- | --- | --- |
|  | **Enrolment** | **Allocation and Baseline** | **Post-allocation** | | | |
| **TIMEPOINT**** | ***-t_1_*** | **0** | ***t_1_*** | ***t_2_*** | ***t_3_*** | ***t_4_*** |
| **ENROLMENT:** |  |  |  |  |  |  |
| **Eligibility screen** | X |  |  |  |  |  |
| **Informed consent** | X |  |  |  |  |  |
| ***Demographic data*** | X |  |  |  |  |  |
| **Allocation** |  | X |  |  |  |  |
| **INTERVENTIONS:** |  |  |  |  |  |  |
| ***Positive Therapeutic Alliance*** |  |  | X |  |  |  |
| ***Usual Treatment*** |  |  | X |  |  |  |
| ***Control Group*** |  |  | X |  |  |  |
| **ASSESSMENTS:** |  |  |  |  |  |  |
| ***Primary Outcomes*** |  |  |  |  |  |  |
| ***Pain Intensity*** |  |  | X | X |  |  |
| ***Specific Disability*** |  |  | X | X |  |  |
| ***Secondary Outcomes*** |  |  |  |  |  |  |
| ***Pain Intensity*** |  |  | X |  | X | X |
| ***Specific Disability*** |  |  | X |  | X | X |
| ***General Disability*** |  |  | X | X | X | X |
| ***Global Perceived Effect*** |  |  | X | X | X | X |
| ***Pain Intensity*** |  |  | X | X | X | X |
| ***Specific Disability*** |  |  | X | X | X | X |
| ***Additional Outcomes*** |  |  |  |  |  |  |
| ***Empathy*** |  |  | X |  |  |  |
| ***Credibility and Expectation*** |  |  | X |  |  |  |

t1: Baseline assessment; t2: Reassessment 1-month after randomization; t3: Reassessment

6-months after randomization; t4: Reassessment 12-months after randomization
