# Supplementary material for: Effectiveness of the addition of therapeutic alliance with minimal intervention in the treatment of patients with chronic, nonspecific low back pain and low risk of involvement of psychosocial factors: a study protocol for a randomized controlled trial (TalkBack trial)
Source: Trials. 2017 Jan 31;18:49. doi: 10.1186/s13063-017-1784-z (PMC5282714; doi:10.1186/s13063-017-1784-z)
Supplement: Additional file 3: — World Health Organization Trial Registration Data Set. (DOC 43 kb) [file 13063_2017_1784_MOESM3_ESM.doc]

**Additional file 3 - World Health Organization Trial Registration Data Set**

| **Data category** | **Information** |
| --- | --- |
| Primary registry and trial identifying number | ClinicalTrials.gov (NCT 02497625) |
| Date of registration in primary registry | July, 2015 |
| Secondary identifying numbers | Ethics Committee (CAAE 44720315.5.0000.5372) |
| Source(s) of monetary or material support | CAPES process number 23633623/35685795865 |
| Primary sponsor | Universidade Cidade de São Paulo |
| Secondary sponsor(s) | Not applicable |
| Contact for public queries | Felipe Ribeiro Cabral Fagundes +551121781565 felipercfagundes@gmail.com |
| Contact for scientific queries | Felipe Ribeiro Cabral Fagundes +551121781565 [felipercfagundes@gmail.com](mailto:felipercfagundes@gmail.com) |
| Public title | Effectiveness of the addition of therapeutic alliance and minimal intervention for low back pain |
| Scientific title | Effectiveness of the addition of therapeutic alliance with minimal intervention in the treatment of patients with chronic non-specific low back pain and low risk of involvement of psychosocial factors: a study protocol for a randomized controlled trial (Talkback Trial) |
| Countries of recruitment | Brazil |
| Health condition(s) or problem(s) studied | Chronic Non-specific Low Back Pain |
| Intervention(s) | Positive Therapeutic Alliance: two sessions of treatment based on an intervention based on treatment guidelines involving guidance and information about low back pain. The session will be structured to increase empathy and therapeutic alliance.  Usual Treatment Group: two sessions of treatment based on an intervention based on treatment guidelines involving guidance and information about low back pain. The sessions will be performed with limited interaction between patient and therapist and the information will be transmitted in a clear and straightforward manner  Control Group: the patients will not receive any treatment. |
| Key inclusion and exclusion criteria | Ages eligible for study: 18 to 80 years Sexes eligible for study: Both Accepts healthy volunteers: No Inclusion criteria: Chronic non-specific low back pain for at least three months and age between 18 and 80 years. Ability to read and write in Portuguese and classified as low risk of having psychosocial factors involvement.  Exclusion criteria: Spine surgery history, serious spine diseases, nerve root compromise, and diseases associated with cognitive impairment determined by medical assessment or pregnants. |
| Study type | Interventional Allocation: Randomized Intervention model: Parallel Assignment Number of Arms: 2 Masking: Single Blind (Outcomes Assessor) Endpoint Classification: Efficacy Study Primary purpose: Treatment |
| Date of first enrolment | September 2015 |
| Target sample size | 222 |
| Recruitment status | Recruiting |
| Primary outcome(s) | Pain and specific disability after one month of randomization. |
| Key secondary outcomes | Pain and specific disability six and 12 months after randomization; and general disability and perceived global effect one, six and 12 months after randomization.  Empathy, credibility and expectations related to treatment will be used as additional outcomes. |
